# RGB-D SLAM with Manhattan Frame Estimation Using Orientation Relevance

**DOI:** 10.3390/s19051050

**Published:** 2019-03-01

**Authors:** Liang Wang, Zhiqiu Wu

**Affiliations:** 1 College of Automation, Faculty of Information Technology, Beijing University of Technology, Beijing 100124, China; s201402158@emails.bjut.edu.cn; 2 Beijing Key Laboratory of Computational Intelligence and Intelligent System, Beijing 100124, China

**Keywords:** SLAM, RGB-D, indoor environment, Manhattan frame estimation, orientation relevance, spatial transformation

## Abstract

Due to image noise, image blur, and inconsistency between depth data and color image, the accuracy and robustness of the pairwise spatial transformation computed by matching extracted features of detected key points in existing sparse Red Green Blue-Depth (RGB-D) Simultaneously Localization And Mapping (SLAM) algorithms are poor. Considering that most indoor environments follow the Manhattan World assumption and the Manhattan Frame can be used as a reference to compute the pairwise spatial transformation, a new RGB-D SLAM algorithm is proposed. It first performs the Manhattan Frame Estimation using the introduced concept of orientation relevance. Then the pairwise spatial transformation between two RGB-D frames is computed with the Manhattan Frame Estimation. Finally, the Manhattan Frame Estimation using orientation relevance is incorporated into the RGB-D SLAM to improve its performance. Experimental results show that the proposed RGB-D SLAM algorithm has definite improvements in accuracy, robustness, and runtime.

## 1. Introduction

Simultaneous Localization and Mapping (SLAM), which aims to acquire the structure of an unknown environment and at the same time estimate the sensor pose with respect to this structure, is an essential task for the autonomy of a robot. It can facilitate a wide range of applications from autonomous robots to virtual and augmented reality. In early SLAM algorithms, many types of sensors, such as rotary encoders, inertial sensors, laser range sensors, and cameras, were employed. Recently, the SLAM algorithms based on the compact Red Green Blue-Depth (RGB-D) sensors, such as Kinect or Xtion, became popular [1,2,3,4,5,6]. This is because RGB-D sensors have the advantages of low price, and appropriate size and weight. More importantly, they can provide direct and dense depth measurements besides the appearance information with the RGB images [7]. Hence, the RGB-D sensors provide opportunities to handle challenges in SLAM systems.

According to the modelling and processing, existing RGB-D SLAM algorithms can be roughly classified into two directories: dense SLAM and sparse SLAM. Newcombe et al. [1,2] firstly introduced dense RGB-D SLAM algorithms in their well-known work, Kinect Fusion. Kinect Fusion can obtain real-time depth measurements and a highly detailed voxel-based map simultaneously. However, their algorithms are only suitable for small workspaces owing to high memory consumption. Moreover, it generally fails when scenes have poor geometric structure. To solve the restricted area problem, Whelan et al. proposed an improved algorithm [3] to densely map large areas in real-time by transforming the voxel grid with sensor pose of each observation. To further improve the efficiency, Keller et al. [4] proposed a point-based fusion representation supporting spatially extended reconstructions with a fused surfel-based model instead of voxel-based representation. In general, dense SLAM algorithms enable good localization and mapping with high quality scene representation [8,9]. However, they are prone to failure in environments with poor structure and time drift. In addition, their computational costs are very high. To some extent some algorithms utilizing sophisticated equipment such as high-end graphics cards can overcome this deficiency. However, their applications’ ranges are constrained.

Instead, sparse RGB-D SLAM algorithms offer a good balance between the computational cost and the quality of pose estimation. Sparse SLAM algorithms are mainly based on the visual odometry, which simply uses visual feature correspondences to compute the motion between the consecutive poses of the RGB-D sensor and then concatenates the pose-to-pose motion. The first RGB-D SLAM algorithm was proposed by Henry et al. [10]. It used features points to estimate sensor poses and then constructed and optimized a graph with nodes representing sensor poses and an edge between two poses being their spatial transformation to refine the localization and mapping. Endres et al. [11] followed the same path and implemented the pose-graph optimization with the G${}^{2}$o framework [12]. Due to its availability, it is very popular. Indeed, sparse RGB-D SLAM algorithms typically run quickly owing to the sensor’s pose estimation based on sparse point features. In addition, such a lightweight implementation ensures a wide range of applications. However, the mapping quality is poor due to limitation of sparse 3D points. More importantly, the mapping result lacks semantic information and there are many repeated and redundant points in the map.

The sparse RGB-D SLAM algorithms have been successful for environments with rich textures. However, they perform poorly and even fail in environments with textureless areas and areas with repetitive textures, which usually exist in indoor scenes with large planar regions [13]. To work well in low-texture environments, researchers begin to show a significant interest in additional high-level geometric information like planar features in recent RGB-D research [14,15,16], and apply them to RGB-D SLAM algorithms [17,18,19]. These SLAM approaches show great improvement in robustness. However, the accuracy still needs to be improved.

Three-dimensional planes in indoor environments, which can be easily extracted from point clouds, are extremely common and are generally relevant. Most indoor environments satisfy the Manhattan World (MW) assumption [20], under which the world consists of a set of orthogonal or parallel planes. Then the environment can be represented by three orthogonal directions, i.e., the Manhattan Frame (MF). The early work of MF estimation was mainly taken RGB images as input, which can be called the RGB image-based methods [21,22]. The RGB image-based methods generally utilize perspective property, such as vanishing line, vanishing point, and orientation map, to estimate the MF with a single RGB image. Recently, the RGB-D sensor is applied to estimate the MF. The corresponding RGB-D image-based methods [15,19] take both color image and depth image as input to compute the MF. In general, RGB image-based methods have poor accuracy and robustness since they mainly depend on information of scene structure in two-dimensional RGB image. RGB-D image-based methods generally perform better than RGB image-based methods [19], since not only the RGB image but also the depth information are explored simultaneously. However, the state-of-the-art of RGB-D image-based methods are still unsatisfactory for real applications, especially in accuracy and speed.

Considering the image noise, image blur, the inconsistency between the depth data and the color image, and especially low-texture (i.e., textureless or repeated texture) planar walls dominating the view of observations, some frames could not be matched to any predecessor yet in existing sparse RGB-D SLAM algorithms. Even if the pairwise spatial transformation can be computed, its accuracy and robustness are poor. On the other hand, most indoor environments follow the MW assumption and the MF can be recovered from a single RGB-D image using orientation relevance [15]. Therefore, a new RGB-D SLAM algorithm is proposed by extending Manhattan Frame estimation (MFE) using orientation relevance to RGB-D image sequence. It first performs MFE using the introduced concept of orientation relevance. Then the pairwise spatial transformation in RGB-D SLAM is computed with the estimated MFE. Finally, the sparse RGB-D SLAM is improved by incorporating MFE using orientation relevance. Experiments validate the proposed algorithm. The contributions of this paper are two-fold: I. A novel algorithm for RGB-D SLAM with MFE using orientation relevance is proposed for low-texture indoor environments. II. It improves the performance of sparse RGB-D SLAM in accuracy and robustness.

The remainder of this paper is organized as follows. Section 2 details the proposed algorithm for RGB-D SLAM with MFE using orientation relevance. Experimental results are presented in Section 3. Finally, we summarize and report future works in Section 4.

## 2. Method

This section presents the proposed RGB-D SLAM method in detail. In the original RGB-D SLAM [11], only point features or all points are used with RANSAC or GICP to estimate the relative spatial transformation between two consecutive observations. Considering the image noise, image blur, and the inconsistency between the depth data and RGB image, some frames could not be matched to any predecessor yet. Even if the pairwise spatial transformation can be computed, its accuracy is not high. It also results in poor robustness or high computational cost. Different from that, the MF of the indoor environment is estimated and used to improve the RGB-D SLAM in the proposed method. In the following, we firstly briefly review the original RGB-D SLAM [11]. Then the algorithm of the Manhattan Frame estimation using orientation relevance is presented. Thirdly, the computation of pairwise spatial transformation with the MFE is presented. Finally, the improved RGB-D SLAM with the Manhattan Frame estimation using orientation relevance is introduced.

### 2.1. Overview of the Original Method

**Figure 1:**
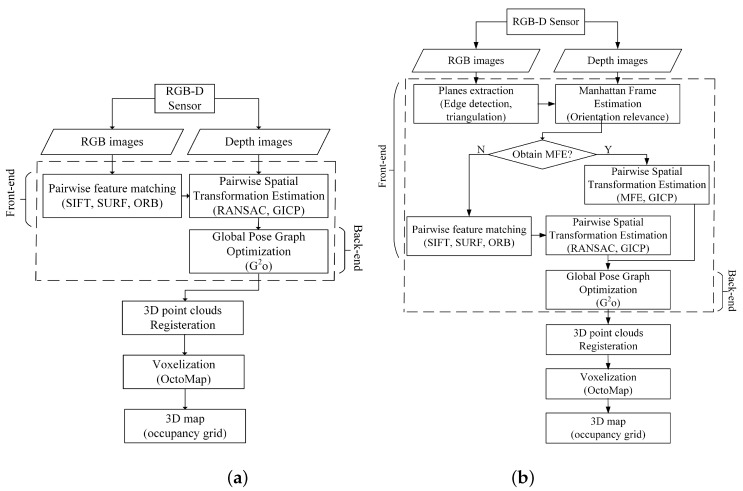
Schematic overview of (**a**) the original Red Green Blue-Depth (RGB-D) Simultaneously Localization And Mapping (SLAM) and (**b**) the proposed RGB-D SLAM.

A schematic overview of sparse RGB-D SLAM is given in Figure 1a [11]. It firstly uses both RGB images and depth data to perform localization and generate the trajectory. Then the mapping is obtained by 3D points registration and voxelization.

The trajectory estimation can be further divided into two parts: the front-end and the back-end. The front-end computes spatial transformations between individual observations, and the back-end computes poses of these observations via a graph-based optimization. In the front-end of the sparse RGB-D SLAM, the RGB image of RGB-D sensor is used to detect key points and extract descriptors. Extracted descriptors of detected key points in two consecutive observations are matched to compute the relative pairwise spatial transformation between two observations using RANSAC. In addition, the depth image of RGB-D sensor makes it possible that dense point clouds of two observations are registered in a common coordinate system using RANSAC or GICP. In the back-end, a non-linear cost function defined on a pose graph [12] is optimized to obtain globally optimal poses of all observations, i.e., the trajectory. After obtaining the trajectory, an occupancy voxel grid map is computed.

### 2.2. Manhattan Frame Estimation Using Orientation Relevance

Due to limitations of RGB-D sensor, the RGB-D SLAM is only applicable for indoor applications. Generally, most man-made indoor environments follow the MW assumption [20], under which the world consists of a set of orthogonal and parallel planes. Three orthogonal directions corresponding to the normal of a set of orthogonal and parallel planes, which are referred to as the MF [15,19], are enough to describe the environment. In RGB-D SLAM, planes in the indoor scene can be detected in each observation. Then candidates of dominant planes can be determined with the constraint of orientation relevance. The MF can be computed by finding the orthogonal dominant planes, which can be described by normal vectors of three orthogonal dominant planes of the scene. It can be further incorporated into RGB-D SLAM to improve the performance of RGB-D SLAM.

Firstly, an edge detection algorithm is run on the input RGB image. Then, end points of detected edges are used to perform 2D Delaunay triangulation to divide the RGB image into several triangles. Next, the triangles are merged according to intensity statistics of pixels in each triangle. Here the intensity statistic, the root mean square error (RMSE) between intensity value of each pixel and the mean intensity of merged area, is taken as measure to merge triangles. Afterwards, the bilateral filter is used to smooth the input depth image. Finally, each plane corresponding to merged triangle in the RGB image, whose area is larger than a threshold, is validated by plane fitting with filtered depth image data. The N (N=9 in our experiments) largest planes are the candidate dominant planes and the normal vector of each candidate plane can be computed with the depth data. These candidate dominant planes are the input of the following MFE using orientation relevance.

An indoor environment satisfying the MW assumption can be denoted by $H=\{P_{1},P_{2},\cdots ,P_{N}\}$, where $P_{n}$ (1 ≤ *n* ≤ *N*, *N* ≥ 3) is one of *N* detected candidate dominant planes. For each pair of two planes $P_{i}$ and $P_{j}$, their relation can be described by the angle between them $\theta_{ij}$. The closer to 0${}^{\circ }$ or $180^{\circ }$ the angle $\theta_{ij}$ is, the nearer two planes $P_{i}$ and $P_{j}$ are parallel. Otherwise, the closer to $90^{\circ }$ the angle $\theta_{ij}$ is, the nearer two planes $P_{i}$ and $P_{j}$ are perpendicular. Most of planes in H are mutually perpendicular or parallel and normal vectors of them can be clustered into three directions. These planes are the dominant planes and three directions are the dominant directions corresponding to the MF. Except for dominant planes, lots of little planar regions existing in indoor environment may have parallel or perpendicular relations. This would lead to error result of MFE. So both the normal direction and area of extracted planar regions should be taken into account. We introduce the concept of orientation relevance of extracted dominant planes, which considers both the area of the projection of extracted planes and the angle between them, to evaluate their geometric relations. The orientation relevance consists of parallel relevance and perpendicular relevance.

The parallel relevance of extracted planes is computed by

(1) $$R_{pa}\left(P_{i}\right)=\sum_{n=1}^{N}A\left(P_{n}\right)\sin(\theta_{in})$$

where $A(P_{n})$ is the area of extracted candidate plane $P_{n}$, $\theta_{in}$ represents the angle between planes $P_{i}$ and $P_{n}$. In fact, $R_{pa}\left(P_{i}\right)$ is the sum of area of all extracted candidate planes’ projection on the plane perpendicular to $P_{i}$. The larger the quantity and area of extracted candidate planes being parallel to $P_{i}$ are, the smaller the value of $R_{pa}\left(P_{i}\right)$ is. Otherwise, the larger the value of $R_{pa}\left(P_{i}\right)$ is.

Similarly, the perpendicular relevance is represented by

(2) $$R_{pe}\left(P_{i}\right)=\sum_{n=1}^{N}A\left(P_{n}\right)\cos(\theta_{in})$$

where $R_{pe}\left(P_{i}\right)$ is the sum of area of all extracted andidate planes’ projection on the plane $P_{i}$. The larger the quantity and area of extracted candidate planes being perpendicular to $P_{i}$ are, the smaller the value of $R_{pe}\left(P_{i}\right)$ is. Otherwise, the larger the value of $R_{pe}\left(P_{i}\right)$ is.

In fact, the parallel relevance and the perpendicular relevance are conflict. To make a compromise, we introduce the term orientation relevance,

(3) $$\begin{array}{cc} R_{o}\left(P_{i}\right) & =f(R_{pe}\left(P_{i}\right),R_{pa}\left(P_{i}\right))\\ & =\sum_{n=1}^{N}A\left(P_{n}\right)\cos(\theta_{in})\sin(\theta_{in})\\ & =\frac{1}{2}\sum_{n=1}^{N}A\left(P_{n}\right)\sin(2\theta_{in}) \end{array}$$

where $\theta_{in}\in \left[0,\frac{\pi }{2}\right]$ is the angle between the plane $P_{i}$ and $P_{n}$. The orientation relevance can reach the minimum in the domain of definition of $\theta_{in}$ when $\theta_{in}=0$ or $\theta_{in}=\frac{\pi }{2}$. In such cases, the relationship between two planes $P_{i}$ and $P_{n}$ is strictly parallel or perpendicular. For indoor environments, one dominant direction may correspond to several parallel dominant planes. Values of the orientation relevance of these parallel dominant planes should be equal in theory. However, they are slightly different from each other in practice due to inevitable noise. Here the dominant direction corresponding to the MF is computed using the dominant plane with the minimal orientation relevance.

(4) $${\tilde{R}}_{o}=min\left\{R_{o}\left(P_{i}\right)\right\}$$

In some cases, it is a planar surface of clutter object rather than a wall that reaches the minimum of orientation relevance. To avoid this case, the area of planar surface is also taken into account,

(5) $${\hat{R}}_{o}=min\left\{R_{o}\left(P_{i}\right)-\lambda A\left(P_{i}\right)\right\}$$

where λ is a coefficient to balance two terms, which usually takes an empirical value of 5000. Then, when the orientation relevance shown in Equation (5) reaches the minimum, the corresponding plane, $P_{D}$, is one of the MW’s dominant planes. The normal of the plane $P_{D}$ corresponds to one axis of the MF.

Then, we determine the other two axes of the MF. Since each detected candidate plane usually differs in position and area, their corresponding values of orientation relevance computed by Equation (5) are different from each other. However, for each of three dominant directions, the corresponding dominant plane should have the minimal orientation relevance among all detected planes sharing this dominant direction. So planes corresponding to the N smallest orientation relevance are initially taken as candidates, where *N* takes 9 in our implementation. Furthermore, the N smallest orientation relevance are sorted in ascending order. Here, the minimal corresponds to the dominant plane $P_{D}$. Additionally, check whether the normal of other N−1 planes is perpendicular to the normal of $P_{D}$ in turn. And take the normal of the first plane whose satisfies the aforementioned condition, $P_{D}^{\prime }$, as the second dominant direction, i.e., the second axis of the MF. Finally, the third dominant direction, i.e., the third axis of the MF can be computed by taking cross product of the first dominant direction and the second dominant direction. By now, three orthogonal directions, i.e., the MF of the indoor environment, are recovered.

### 2.3. Computation of Pairwise Spatial Transformation with the MFE

Once the MF of one observation is computed, it can be used to compute the pairwise spatial transformation of current pose relative to its previous one, and then be incorporated into the RGB-D SLAM to improve its performance.

The MF can be described by unit normal vectors of dominant orthogonal planes. Generally, two unit normal vectors of two orthogonal dominant planes are enough. For example, the unit normal vector of two orthogonal dominant planes is denoted by $m_{1}$ and $m_{2}$ respectively. They correspond to two orthogonal directions of the MF. The third direction of the MF can be computed by

(6) $$m_{3}=m_{1}\times m_{2}$$

Then the MF of current observation can be described by unit normal vectors of three orthogonal dominant planes

(7) $$M_{1}=\left[m_{1}\;m_{2}\;m_{3}\right]$$

Similarly, the MF of the previous observation can be described as

(8) $$N_{1}=\left[n_{1}\;n_{2}\;n_{3}\right]$$

For an RGB-D SLAM application, the MF of the indoor scene is fixed. However, there are relative translation and rotation between two consecutive observations for RGB-D sensor, which make the computed MFs $M_{1}$ and $N_{1}$ are different in two local coordinate systems of two observations. The spatial transformation between two consecutive observations in RGB-D SLAM, ***T***, consists of ***R*** and ***t***.

(9) $$T=\left[\begin{array}{cc} R & t\\ 0^{T} & 1 \end{array}\right]$$

where ***R*** and ***t*** is the relative rotation matrix and translation vector between two observations respectively. The relative rotation ***R*** between two observations can be computed with the MFs estimated in local coordinate system of two observations.

(10) $$R\cdot m_{i}=n_{i}\;\left(s.t.R^{T}R=I\;and\;det\left(R\right)=1\right)\;\left(i=1,2,3\right)$$

As Equation (10) shows, the corresponding MFs of two observations can provide 9 equations to compute unknowns in ***R***. However ***R*** is a unit orthogonal matrix, some constraints, such as $R^{T}R=I$ and det(R)=1 (where ***I*** is an identity matrix, det(·) denotes the determinant of a matrix), should be satisfied, which results in a complex constrained optimization problem. For each pair of consecutive observations, ***R*** can be firstly computed by linearly solving equation system $R\cdot m_{i}=n_{i}\;\left(i=1,2,3\right)$, and then enforced the constraints $R^{T}R=I$ and det(R)=1. Once the rotation matrix ***R*** is obtained, the point cloud corresponding to the current observation can be transformed to the local coordinate system of the previous observation using the obtained ***R***. Then the translation vector ***t*** can be computed by GICP with the transformed point cloud of current observation and the point cloud of previous observation.

The spatial transformation between each pair of consecutive observations, T, can be further optimized by bundle adjustment by solving the following unconstrained optimization problem

(11) $$e=\underset{\xi }{min}\frac{1}{2}\sum_{i=1}^{N}∥p_{i}-exp\left(\xi^{\Lambda }\right)q_{i}{∥}_{2}^{2}$$

where $p_{i}$ and $q_{i}$ is the 3D point in the point cloud of previous observation and that of current observation respectively, $\xi =\left[\begin{array}{c} \rho \\ \phi \end{array}\right]\in R^{6}$ is the Lie algebraic representation of transformation and the relation between the spatial translation and the its Lie algebraic representation follows

(12) $$T=exp\left(\xi^{\Lambda }\right)=\left[\begin{array}{c} exp(\phi^{\Lambda })\;J\rho \\ 0\;\;\;1 \end{array}\right]$$

where

(13) $$exp\left(\phi^{\Lambda }\right)=exp\left(\theta a^{\Lambda }\right)=cos\theta I+\left(1-cos\theta \right)aa^{T}+sin\theta a^{\Lambda }$$

(14) $$J=\frac{sin\theta }{\theta }I+\left(1-\frac{sin\theta }{\theta }\right)aa^{T}+\frac{1-cos\theta }{\theta }a^{\Lambda }$$

(15) $$\theta =arccos\frac{tr(R)-1}{2}$$

(16) Ra=a

(17) t=Jρ

The Lie algebra $\mathrm{se}\left(3\right)=\left\{\xi =\left[\begin{array}{c} \rho \\ \phi \end{array}\right]\in R^{6},\rho \in R^{3},\phi \in R^{3},\xi^{\Lambda }=\left[\begin{array}{c} \phi^{\Lambda }\;\rho \\ 0^{T}\;0 \end{array}\right]\in R^{4\times 4}\right\}$, which corresponds to the tangent space of the Lie group $SE\left(3\right)=\{T=\left[\begin{array}{cc} R & t\\ 0 & 1 \end{array}\right]\in R^{4\times 4}|R\in R^{3\times 3},R^{T}R=I,det\left(R\right)=1,t\in R^{3}\}$, describes the local derivatives. Here we use the Lie algebraic representation to optimize the spatial transformation. On one hand, with the Lie algebra, the obtained unconstrained optimization problem is relatively easier to solve than the corresponding constrained one. On the other hand, the Lie algebra representation makes the computation of derivatives easier during the optimization process. The unconstrained optimization problem Equation (11) can be solved by the Gaussian-Newton method or Levenberg-Marquardt algorithm. Then the pairwise spatial transformation ***T*** is obtained.

### 2.4. Improved RGB-D SLAM

Considering the RGB-D SLAM is only applicable for indoor applications and the MF of the indoor scene is fixed, the MF can be used as a reference to compute the pairwise spatial transformation. So a new algorithm of RGB-D SLAM shown in Algorithm 1 is proposed, in which the aforementioned pairwise spatial transformation computation with MFE using orientation relevance is incorporated into the original RGB-D SLAM [11] to improve its performance as shown in Figure 1b.

**Algorithm 1** RGB-D SLAM with MFE Using Orientation Relevance
**Input:** RGB-D sequences
**Output:** Trajectory of RGB-D sensor and reconstructed environment.
Step 1. Extract planes from the RGB image using edge detection and triangulation of end points of detected edges.Step 2. Estimate Manhattan Frame using orientation relevance with dominant planes determined by cross validation on depth information and planes extracted from RGB image.Step 3. Determine whether the MFE is available. If it’s available, compute the pairwise spatial transformation with MFE and GICP, and then jump to Step 5. Otherwise, go to Step4.Step 4. Compute the pairwise spatial transformation following the routine of the original RGB-D SLAM.Step 5. Optimize the trajectory.Step 6. Registrate 3D point clouds.Step 7. Voxelize the registrated 3D point clouds.Step 8. Reconstruct the 3D map.**return** Trajectory and 3D map.


Different from conventional RGB-D SLAM, which uses correspondences of feature points to compute the pairwise spatial transformation between two consecutive observations, the proposed RGB-D SLAM exploits the information of dominant planes. This makes the computation of pairwise spatial transformation more robust and accurate. In addition, in conventional RGB-D SLAM, the estimated trajectory is usually divided into several fragments due to the failure of feature matching of detected key points in pairwise spatial transformation computation caused by image noise, image blur and the inconsistency between the depth data and RGB image, which increases the complexity of the optimization problem of the back-end of RGB-D SLAM. Whereas, the proposed improved RGB-D SLAM is more robust and can reduce the number of trajectory fragments which makes the corresponding optimization problem more easily and rapidly converge to the global optimum.

## 3. Experiments

**Figure 2:**
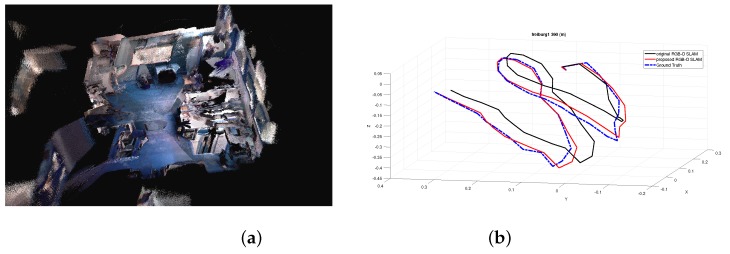
Experimental results of the proposed RGB-D SLAM with sequence fr1/360. (**a**) Mapping results in the form of volumetric 3D model. (**b**) Estimated trajectories.

**Figure 3:**
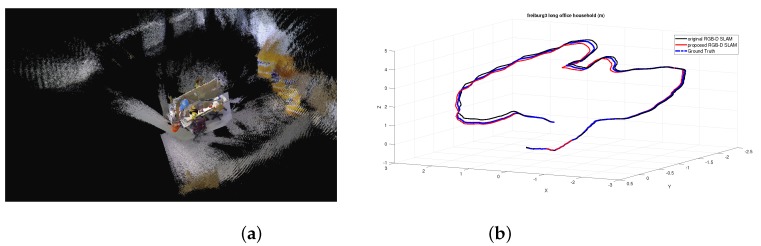
Experimental results of the proposed RGB-D SLAM with sequence fr3/long_office_household. (**a**) Mapping results in the form of volumetric 3D model. (**b**) Estimated trajectories.

**Figure 4:**
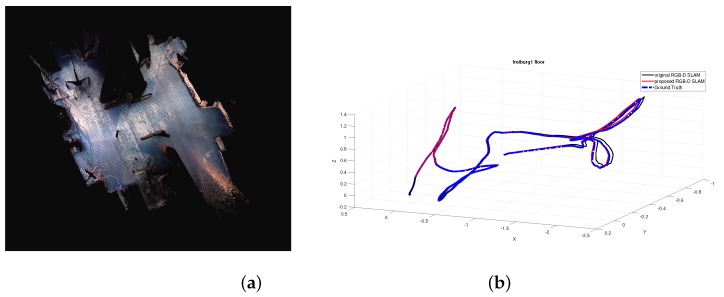
Experimental results of the proposed RGB-D SLAM with sequence fr1/floor. (**a**) Mapping results in the form of volumetric 3D model. (**b**) Estimated trajectories.

To validate the proposed RGB-D SLAM algorithm, some experiments are performed on a computer with an AMD Phenom II X6 1055T 3.36GHZ CPU and 8GB RAM with the RGB-D dataset and benchmark [23], which provides a dataset of RGB-D sequences from the Kinect and synchronized ground truth pose estimates from the motion capture system. These sequences are captured in a typical indoor environment. Furthermore, the benchmark provides an evaluation tool to compute the RSME. For the convenience of comparison, we use the benchmark tool to evaluate the proposed algorithm. To make a comparison, experiments using the original RGB-D SLAM [11] without the MF estimation are also performed. To show the comparison results in different scenes and different complexity of motion, experiments of 3 sequences are reported here. Critical details of 3 sequences are shown in Table 1. The structure and appearance of each scene can be seen in the following mapping results in the form of volumetric 3D model shown in Figure 2a, Figure 3a, and Figure 4a, respectively.

The fr1/360 scene is a typical indoor office which includes walls, floor, table and clutters. Table 2 shows the trajectory results of original RGB-D SLAM [11] and the proposed improved RGB-D SLAM. To make a comparison, results of RGB-D SLAM with RMFE algorithm are also reported in Table 2, which are directly cited from [19]. As can be seen from this table, the proposed improved RGB-D SLAM outperforms the original RGB-D SLAM and RGB-D SLAM with RMFE in RMSE of translation, RMSE of rotation and runtime. The most obvious improvement is in runtime, which dramatically drops from 145 s for the original algorithm to 100 s for the improved algorithm. It has about 31% relative improvement (RI) with respect to the corresponding parameter of the original RGB-D SLAM. The RMSE of translation drops from 0.103 m to 0.082 m, which has about 20% RI. The RMSE of rotation drops from 3.41 degrees to 3.10 degrees, which has about 9% RI. Results of estimated trajectory for fr1/360 are shown in Figure 3a. It can be seen that the trajectory estimated by the proposed algorithm is much closer to the ground truth than that of the original RGB-D SLAM. We could not find the source code and detailed parameters of RGB-D SLAM with RMFE. In fairness, we do not show the estimated trajectory of the RGB-D SLAM with RMFE implemented by us to make comparisons since results of RMFE [19] implemented by us are inferior to MFE using orientation relevance as shown in Ref. [15].

To further validate the proposed method, experiments are also performed on sequence of fr3/long_office_household and fr1/floor. Considering reasons mentioned above and results shown in Table 1 that the proposed method outperforms the RGB-D SLAM with RMFE, results of the RGB-D SLAM with RMFE implemented by us are not reported here. The sequence of fr3/long_office_household mainly focuses on an office table and its indoor environment. The office table is in the center of this scene, which is surrounded by white walls. Since the range of the scene is so large that the wall and floor far from the table are out of the measurement range of RGB-D sensor, there are some areas with lots of missing data. Results of estimated trajectory of fr3/long_office_household are shown in Figure 3b. As can be seen, the trajectory estimated by the proposed method is much closer to the ground truth than that of the original RGB-D SLAM. From Table 3 we can see that the runtime drops 211 s which results in about 29% RI, the RMSE of translation drops 0.03 m which brings in about 37% RI, and the RMSE of rotation drops 0.11 degrees which brings in about 7% RI. The sequence of fr1/floor mainly focuses on the indoor floor which is marked with blue color, and there is some clutter on the floor. The results of the estimated trajectory for fr1/floor are shown in Figure 4b, where the trajectory estimated by the proposed method is much closer to the ground truth than that of the original RGB-D SLAM. As can be seen from Table 4, the runtime drops 86 s which brings in about 18% RI, the RMSE of translation drops 0.006 m which results in about 10% RI, and the RMSE of rotation drops 0.03 degrees which results in about 1% RI. It is noted that since the scene range becomes larger, and the visual difference between trajectories becomes slighter in comparison with Figure 2b. However, improvements brought by the proposed method are obvious.

From experimental results, we can see that the proposed method consistently outperforms the original RGB-D SLAM. The improvement brought by the proposed RGB-D SLAM on sequence of fr3/long_office_household and fr1/360 are larger than that on sequence of fr1/floor. The reason is mainly because that the focus of sequence of fr1/floor is floor and images containing two or more orthogonal dominant planes are relatively less. Furthermore, it is hard to find enough orthogonal dominant planes to perform MFE in these sequences. As shown in Figure 1b, pairwise spatial transformation estimation with MFE using orientation relevance will fail and conventional routine of the original RGB-D SLAM, which performs pairwise spatial transformation estimation with detection and matching of feature points and registration of 3D point clouds with RANSAC scheme, will function in this case. So in the worst case where the the MW assumption does not hold, the proposed method degrades to the original RGB-D SLAM. Fortunately, the conventional routine of the original RGB-D SLAM is fully functioning in most of these cases since clutter in a small measurement range provide rich texture. So although the trajectory segments of the degraded proposed method coincide with those of the original method in the above experiments, rich textures ensure that the trajectory segments of the original RGB-D SLAM are very close to the ground truth as seen in Figure 3b and Figure 4b. When there are a few low-texture walls corresponding to two or more orthogonal dominant planes in observations of RGB-D SLAM, the performance of the original RGB-D SLAM will degrade. While the proposed method fulfils its function and performs well. In summary, the proposed RGB-D SLAM can bring in obvious improvements in runtime and accuracy of trajectory in comparison with the original RGB-D SLAM and RGB-D SLAM with RMFE. The reasons may be as follows: (1) Using MF estimation with orientation relevance instead of conventional detection and matching of feature points with RANSAC scheme to compute the pairwise spatial transformation in the front-end of RGB-D SLAM can bring in performance improvement. (2) The optimization problem of the back-end of RGB-D SLAM becomes easier since the aforementioned reason leads to a good initialization and less trajectory fragments, which also improves the performance and reduces runtime. Experiments also show that the proposed method is suitable for sequences with different duration, range, and motion velocity. Hence, the proposed method is valid and reliable.

## 4. Conclusions

A new method of RGB-D SLAM is proposed, which computes the pairwise spatial transformation with the MFE using orientation relevance instead of the conventional routine of the original RGB-D SLAM, which uses detection and matching of point correspondences and registration of 3D point clouds with the RANSAC scheme. It can overcome the deficiency of the original RGB-D SLAM that some observations of RGB-D sensor could not be matched to any predecessor due to image noise, image blur, inconsistency between the depth data and the RGB image, and especially low-texture (i.e., textureless or repeated texture) planar walls dominating the view of observations. Experiments on an open dataset benchmark validate the proposed method. It can bring in obvious improvements in runtime and accuracy of trajectory in comparison with the original RGB-D SLAM and RGB-D SLAM with RMFE. In the future, we will further improve the proposed method to be suitable for real-time applications and extend it to more complex indoor environments such as the Atlanta world [24]. We will also further improve the RGB-D SLAM to be applicable to dynamic environments.

## Figures and Tables

**Table 1:** Details of sequences from the Red Green Blue-Depth (RGB-D) Simultaneously Localization And Mapping (SLAM) dataset [23].

Sequence	Frames	Duration (s)	Length (m)	Avg. Trans. Velocity (m/s)	Avg. Rot. Velocity (${}^{\circ }$/s)	Range (m${}^{3}$)
fr1/360	745	28.69	5.82	0.21	41.60	0.54 × 0.46 × 0.47
fr3/long_office _household	2585	87.09	21.45	0.25	10.19	5.12 × 4.89 × 0.54
fr1/floor	1214	49.87	12.57	0.258	15.07	2.30 × 1.31 × 1.58

**Table 2:** Trajectory results of RGB-D SLAM with fr1/360 sequence.

Method	Translation		Rotation		Runtime	
	RMSE (m)	RI	RMSE (${}^{\circ }$)	RI	(s)	RI
original method [11]	0.103	−	3.41	−	145	−
method with RMFE [19]	0.107	−3.9%	3.37	1.2%	112	23%
proposed method	0.082	20%	3.10	9%	100	31%

**Table 3:** Trajectory results of RGB-D SLAM with fr3/long_office_householdsequence.

Method	Translation		Rotation		Runtime	
	RMSE (m)	RI	RMSE (${}^{\circ }$)	RI	(s)	RI
original method [11]	0.082	−	1.63	−	722	−
proposed method	0.052	37%	1.52	7%	511	29%

**Table 4:** Trajectory results of RGB-D SLAM with fr1/floor sequence.

Method	Translation		Rotation		Runtime	
	RMSE (m)	RI	RMSE (${}^{\circ }$)	RI	(s)	RI
original method [11]	0.061	−	2.72	−	488	−
proposed method	0.054	11%	2.69	1%	402	18%

## Abbreviations

The following abbreviations are used in this manuscript:

SLAM	Simultaneously Localization And Mapping
RGB-D	Red Green Blue-Depth
3D	three Dimensional
MW	Manhattan World
MF	Manhattan Frame
MFE	Manhattan Frame Estimation
RANSAC	RANdom SAmple Consensus
GICP	Generalized Iterative Closest Point
RMFE	Robust Manhattan Frame Estimation
RMSE	Root Mean Square Error
RI	Relative Improvement
